# Assessment of Arteriovenous Fistula Maturation in Hemodialysis Patients with Persistently Positive Antiphospholipid Antibody: A Prospective Observational Cohort Study

**DOI:** 10.3390/life15020168

**Published:** 2025-01-24

**Authors:** Maxime Taghavi, Lucas Jacobs, Saleh Kaysi, Yves Dernier, Edouard Cubilier, Louis Chebli, Marc Laureys, Frédéric Collart, Anne Demulder, Marie-Hélène Antoine, Joëlle Nortier

**Affiliations:** 1 Nephrology and Dialysis Department, Brugmann University Hospital, Université Libre de Bruxelles (ULB), 1020 Brussels, Belgium; 2 Laboratory of Experimental Nephrology, Faculty of Medicine, Université Libre de Bruxelles (ULB), Erasme Campus, 1070 Brussels, Belgium; 3 Vascular Surgery Department, Brugmann University Hospital, Université Libre de Bruxelles (ULB), 1020 Brussels, Belgium; 4 Radiology Department, Brugmann University Hospital, Université Libre de Bruxelles (ULB), 1020 Brussels, Belgium; 5 Laboratory of Hematology and Hemostasis, Brugmann University Hospital, Université Libre de Bruxelles (ULB), 1020 Brussels, Belgium

**Keywords:** arteriovenous fistula, maturation, antiphospholipid antibodies, hemodialysis

## Abstract

Background: Arteriovenous fistula (AVF) is the preferred vascular access option for hemodialysis (HD). The latter requires a remodeling process called maturation that can take up to 3 months. Maturation failure is a frequent complication associated with significant morbidity. The prevalence of antiphospholipid antibody (aPL) positivity in HD patients is high and may result in thrombosis of the vascular access. Recently, aPL persistent positivity has been associated with AVF maturation failure in a retrospective study including 116 patients. Methods: We are conducting an observational prospective cohort study aiming to evaluate this association. Included patients are planned for AVF creation, aged over 18 years old, and have an interpretable aPL assay confirmed at 12 weeks and without any other innate or acquired thrombophilia or inflammatory disease. Primary endpoints will be the evaluation of AVF maturation clinically and by ultrasound. Secondary endpoints will focus on clinical outcomes other than AVF maturation (i.e., primary patency, thrombosis or stenosis, bleeding and hemodialysis adequacy parameters). Conclusions: This prospective observational cohort study aims to examine the possibly causative link between aPL persistent positivity and AVF maturation failure. This study was registered on ClinicalTrials.gov (ID number: NCT06112821).

## 1. Introduction

End-stage kidney disease (ESKD) is characterized by the need to initiate renal replacement therapy to control clinical, biological, and hemodynamic complications. Renal transplantation, peritoneal dialysis, and hemodialysis (HD) are currently the treatment options for ESKD. The Kidney Disease Outcomes Quality Initiative (KDOQI) guidelines advocate for the use of native arteriovenous fistula (AVF) as the primary option for establishing HD vascular access in patients with ESKD [1]. Arteriovenous fistula is associated with lower morbidity and mortality compared to arteriovenous grafts or the placement of a HD central venous catheter [2].

AVF creation consists of performing a surgical or endovascular anastomosis of a native artery to an adjacent native vein of the non-dominant superior limb. AVF creation encompasses the outflow vein structural changes characterized by an increased efferent vein diameter, thickness and blood flow [3,4]. This process, usually called “maturation”, takes place within 6 weeks to 3 months and is crucial for routine AVF puncture in HD [5]. Maturation failure of a native AVF is a frequent complication and is characterized by the absence of, or a delay of, maturation, defined either clinically or by using Doppler ultrasound (US) [3,6].

Antiphospholipid syndrome (APS) is an autoimmune disease characterized by the persistent positivity of at least one antiphospholipid antibody (aPL). It is an acquired thrombophilia that affects both the arterial and the venous vasculature. New classification criteria were released in 2023, based on a scoring system [7]. With respect to laboratory domains, the following antibodies are included: lupus anticoagulant (LA); the IgG or IgM anti-cardiolipin antibody (aCL); and the IgG or IgM anti β2 glycoprotein I (aβ2-GPI). Confirmation of a positive assay is mandatory at 12 weeks, leading to confirming the biological criterion. In the absence of any clinical criteria, aPL persistent positivity does not allow for the diagnosis of APS. It is interesting to note that the 2023 ACR/EULAR classification criteria no longer consider isolated positivity of IgM aCL and/or IgM aβ2-GPI [either at moderate titers (40–79 units/mL) or high titers (>80 units/mL)] as sufficient in order to classify a patient as having APS [7].

Although ESKD is rare in APS, the prevalence of persistent aPL positivity is higher in HD patients compared to the general population, and can reach more than 35% [8]. The reason for such a high prevalence of aPL positivity in HD is not well known. Several hypotheses have been proposed such as (a) molecular mimicry as a response to the exposure to microorganisms, endotoxins or HD membranes, or (b) a response to oxidation (i.e., cross-reactive immunoglobulins against epitopes of oxidized lipids) [8]. Nevertheless, aPL positivity has been inconsistently associated with AVF thrombosis [8,9,10,11]. Also, aPL positivity has been rarely associated with AVF stenosis [12] and intrastent restenosis [13]. Recently, our group published the first report of a significant association between aPL persistent positivity, and native AVF maturation failure as defined by US in a retrospective study including 116 HD patients [14]. The aPL positivity was a strong predictor of AVF maturation failure in multivariate analysis.

The aim of the present study is to confirm this association in a prospective cohort. The primary outcome of the study is to examine the possibly causative link between aPL persistent positivity and the occurrence of AVF maturation failure defined by the clinical approach combined with US.

Secondary outcomes will focus on clinical outcomes other than AVF maturation: functional primary patency and primary patency, time to AVF complications, AVF thrombosis or stenosis rates, bleeding complications rates and HD adequacy parameters.

In addition, in order to better understand the underlying pathophysiological mechanism of AVF maturation failure in aPL positive patients, we will perform blood analyses as well as an in vitro study using cultured immortalized human microvascular endothelial cells type 1 (HMEC-1).

## 2. Materials and Methods

### 2.1. Design and Objectives

This is a monocentric observational prospective cohort study that aims to recruit a cohort of 100 newly created native AVF in CKD patients with a previous aPL profile determination before AVF creation and HD initiation.

The sample size calculation was based on the assumption that the expected maturation failure rate in the general population is 50%, and is 70% in the aPL positive group. With alpha = 0.05, 1-Beta = 90%, and a two-sided test, 96 patients are required to detect a significant difference. Thus, we plan to enroll and analyze 100 patients.

Surgical AVF creation is performed as per standard of care without procedural variations between the aPL+ and control groups, ensuring homogeneity in the approach. No perioperative treatments or clinical assessments are specified by the study protocol.

**Figure 1:**
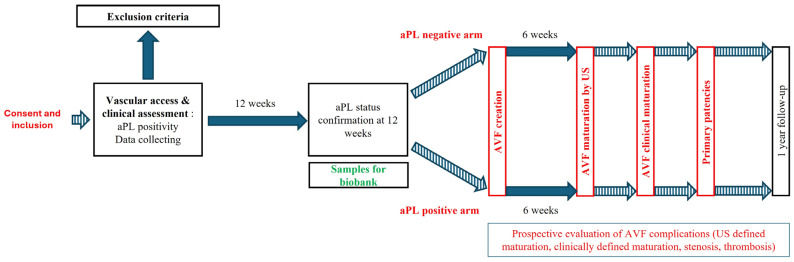
Schematic view of the study protocol timeline. Full arrows represent defined duration (i.e., 12 weeks for aPL confirmation assay, or 6 weeks for AVF maturation evaluation), and dotted arrows represent undefined periods.

Institutional Review Board authorization was obtained from the Brugmann University Hospital Ethics Committee (Reference number: CE2022/113), in accordance with the Declaration of Helsinki. Written informed consent is obtained from patients before any inclusion procedure. This study was registered on ClinicalTrials.gov (ID number: NCT06112821), and the study was started on August 2023. The study protocol is illustrated in Figure 1.

### 2.2. Participants

Patients are eligible for inclusion if they fulfill the following criteria: patients with chronic kidney disease (CKD) on stage G3b to G5 who have not yet started HD, aged from 18 to 75 years old, who are planned for a first native AVF surgical creation as decided by a multidisciplinary team comprising nephrologists and vascular surgeons, irrespective of previous aPL status.

Exclusion criteria are the following: ESKD patients switching from peritoneal dialysis to HD, AVF creation not feasible surgically or technically, endovascular AVF, innate or acquired thrombophilia other than APS, active neoplasia, aPL assays not relevant (because of anti-vitamin K treatment, inflammatory state, absence of confirmation assay, etc.).

### 2.3. Study Groups

We classified patients according to the new APS 2023 ACR/EULAR classification criteria. Patients are undergoing a clinical and laboratory assessment, allowing for the evaluation of criteria clustered into six clinical domains (macrovascular venous thromboembolism, macrovascular arterial thrombosis, microvascular, obstetric, cardiac valve, and hematologic) and two laboratory domains [7]. The patients are divided into the following two groups:

aPL positive arm: gathering all patients with aPL persistent positivity (laboratory domains), including:

APS: patients with at least three points in both clinical and laboratory criteria according to the 2023 ACR/EULAR classification criteria for APS.

aPL carriers without APS: patients with at least three points in laboratory criteria but fewer than three points in clinical criteria

aPL negative arm: gathering patients with one (or more) negative aPL assay(s), or patients with an initial positive assay but showing a negative control assay at the 12-week follow up.

Given the heterogeneity of the aPL positive arm, the two subgroups (APS and aPL carriers) will be analyzed to explore AVF maturation failure, thrombosis, stenosis and patency.

### 2.4. Follow Up

Participants will be followed clinically for 1 year after the date of AVF creation (Figure 1. Study protocol).

### 2.5. Data Collection

In order to limit aPL false positivity, we aim to evaluate aPL profile before AVF creation and HD initiation. Antiphospholipid antibody assays are collected at the time of registration in the study. If not performed, the 12-week follow-up confirmation assay is performed at the time of registration, and before AVF creation.

Lupus anticoagulant (LA) positivity is assessed by using a three-step diagnostic procedure: screening, mix and confirmation procedures using diluted Russell’s viper venom (dRVVT-Siemens^®^, Muenchen Germany) and Silica Clotting time (SCT-Werfen^®^, Barcelona, Spain). Results are expressed as screening-to-confirmation ratios. LA is confirmed if one of the two functional coagulation assays (dRVVT or SCT) is positive in terms of screening-to-confirmation ratio, using a citrated plasma sample (3.2%). The determination of IgG/IgM aCL and IgG/IgM aβ2GPI is performed by a chemiluminescence immunoassay (HemosIL Acustar aCL IgM/IgG Kit and aβ2GPI IgM/IgG kit- Werfen^®^). According to the standards of our laboratory, the results are interpreted as positive (>the 99th percentile) or negative when the IgG or IgM titers were, respectively, >20 U/mL or ≤20 U/mL. Baseline characteristics will be collected at the time of AVF surgical creation and are presented in Table 1.

### 2.6. Research Samples of Plasma and Sera

Plasma and sera samples are obtained from patients before AVF surgical creation and stored for further determination in the validated biobank MARENVU, at the laboratory of experimental nephrology, Faculty of Medicine, ULB.

For plasma preparation, we used 5 mL commercially available 3.2% citrate-treated tubes (light blue tops) from Becton Dickinson (BD) Vacutainer^®^ (Franklin Lakes, NJ, USA) Cells were removed from plasma by centrifugation for 15 min at 2000× *g* in order to deplete platelets in the plasma sample. The resulting supernatant (plasma) was then immediately apportioned into 0.5 mL aliquots using a clean polypropylene tube using a Pasteur pipette.

For serum preparation, we used a red topped 5 mL available tubes from Becton Dickinson (BD) Vacutainer^®^ for collection of whole blood. We waited 30 min at room temperature, in order to allow the blood clot formation. Sera was separated from blood by centrifuging at 1000–2000× *g* for 10 min in a refrigerated centrifuge. The resulting supernatant (serum) was then immediately apportioned into 0.5 mL aliquots using a clean polypropylene tube using a Pasteur pipette. Regarding storage and manipulation, plasma and sera are not analyzed immediately. All aliquots are stored and transported at −80 °C. Specific care is taken in order to avoid freeze–thaw cycles. When defrosted, sera are maintained at 2–8 °C while being handled. Samples which are hemolyzed, icteric or lipemic are excluded.

### 2.7. Patient Outcomes

Prospective follow-up is performed with a follow up of one year after AVF creation. AVF primary and secondary outcomes are listed in Table 2.

### 2.8. Statistical Analyses

The total expected sample size is assumed to be a minimum of 100 patients based on an average recruitment rate of 2 patients/month. Analyses will describe AVF clinical outcomes for patients with and without persistent aPL positivity, potential predictors of patient outcome. Analyses will use all available data, regardless of final sample size.

Data will be expressed as mean ± standard deviation (SD) for variables with a normal distribution or as median ± interquartile range (P25; P75). Student’s t-test will be used to compare the means of the quantitative variables following a normal distribution by group. The Mann–Whitney Wilcoxon test will be used to study the variation between two groups of variables following an asymmetric distribution. The significance level of the tests was 0.05 with odds ratios and 95% confidence intervals. Multivariate models will be performed through logistic regression for variables showing statistically significant differences between groups.

We will employ Kaplan–Meier survival analysis to estimate the probability of AVF primary patency and functional primary patency over time.

### 2.9. Future Analyses

To better understand the underlying pathophysiological mechanism of AVF maturation failure in aPL positive patients, we will perform blood analyses as well as an ex vivo study using cultured immortalized human microvascular endothelial cells type 1 (HMEC-1).

Blood samples:

The following markers of extracellular matrix (ECM) degradation will be assessed: matrix metalloproteases (MMP): MMP-2, MMP-9, tissue inhibitor of metallo-proteinase-1 (TIMP-1) and the ratio of MMP-2 and TIMP-1 will be evaluated and interpreted as a predictor of AVF maturation as prior described [15].

In Vitro study

HMEC-1 will be exposed to sera of aPL-positive patients and aPL-negative patients as controls. We will perform a comprehensive set of analyses across various biological process potentially implicated in the pathophysiology of AVF maturation failure. We will assess coagulation markers, inflammatory and leukocyte trafficking markers, complement system deposition and vascular tone, and metabolism-related markers and extracellular matrix degradation.

## 3. Discussion

AVF maturation failure is a frequent complication that occurs in more than half of cases after surgical creation of the AVF, and often requires the use of intervention [16], possibly leading to significant morbidity and mortality [1,2,16]. Therefore, a better understanding of the risk factors of AVF maturation failure is of importance in order to identify prophylactic or therapeutic strategies.

In the setting of persistent aPL positivity, AVF maturation failure could be related to early AVF thrombosis. Also, APS has been associated with endothelial dysfunction and intimal hyperplasia with stenotic lesions in some aPL-related manifestations such as APS nephropathy [17,18]. We recently described the association between aPL persistent positivity, and native AVF maturation failure defined by US. aPL positivity was a strong predictor in multivariate analysis; however, this association was independent of AVF stenosis or thrombosis during the maturation process [14]. These findings might suggest that aPL persistent positivity could be responsible for the inability of the efferent vein vasodilatation and remodeling leading to AVF maturation failure. However, these findings might also reflect the statistical artifact of combining two outcomes (i.e., AVF rachial artery blood flow and outflow vein diameter). Therefore, in order to confirm these results, we propose a prospective observational cohort study that aims to recruit a cohort of newly created native AVFs with a previous aPL determination and 12-week confirmation assays, with longitudinal clinical and US follow-ups. Analyses will describe AVF clinical outcomes, in terms of maturation, in patients with and without persistent aPL positivity, potential predictors of patient outcome. The results could have a significant clinical impact and pave the way for new exploratory or confirmatory studies.

Furthermore, we will further evaluate the pathophysiological mechanisms implicated in AVF maturation failure in the setting of aPL persistent positivity. After AVF creation, the efferent vein is exposed to oxygen-rich blood flow, but also to high shear stress, and high pressure. These changes will lead to morphological changes in both the arterial and venous wall (i.e., thickening and dilatation) [15]. Efferent vein remodeling is mediated by both venous endothelium and adventitia that sensor the hemodynamic forces change [19,20]. Initial phase implies the increase in MMP-2 and MMP-9, and the ratio of MMP-2 and TIMP-1 has been reported as a predictor of AVF maturation [15]. Also, other elastases such as cathepsin S and K are upregulated in the AVF, suggesting that elastin degradation is crucial in order to enhance efferent vein remodeling, by degrading the internal elastic lamina and basement membrane [21,22]. Also, numerous growth factors and cytokines play a crucial role in AVF maturation, particularly by regulating ECM synthesis or degradation, or by controlling cell proliferation and migration [15]. Interestingly, the expression of the MMP-2/-9 is decreased in the setting of aPL positivity, suggesting that MMPs can be involved in AVF maturation failure in the setting of aPL positivity [23].

**Figure 2:**
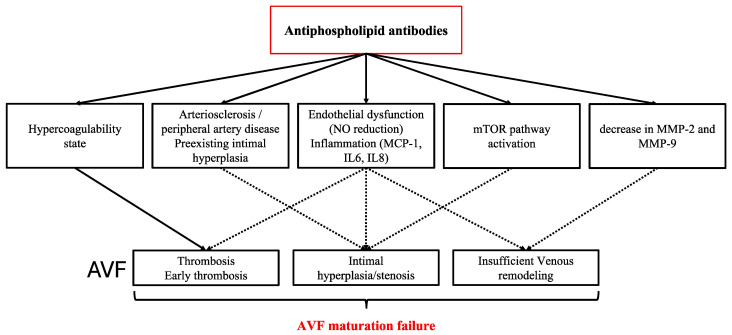
Pathophysiological hypothesis of aPL-associated AVF maturation failure. The first line represents the pathways implicated in the pathophysiology of APS and the second line represents the presentation of AVF maturation failure (either in the presence of thrombosis, stenosis or because of insufficient venous remodeling). The dashed arrows represent pathophysiological hypotheses that have not been studied in the literature, while the solid arrows represent associations described in the literature. MCP-1: Monocyte Chemotactic Protein 1, MMP: matrix metalloproteases, mTOR: mammalian Target of Rapamycin, NO: nitric oxide, TIMP-1: tissue inhibitor of metallo-proteinase-1.

Because aPL persistent positivity was associated with AVF maturation failure in the absence of stenosis or thrombosis, we hypothesize that endothelial dysfunction—through impaired NO release and/or decreased levels of MMPs or elastases—could be responsible for the inability of the efferent vein vasodilatation and remodeling leading to AVF maturation failure (Figure 2).

This study has the potential to significantly impact the clinical management of hemodialysis patients by identifying persistent antiphospholipid antibody (aPL) positivity as a predictive biomarker for arteriovenous fistula (AVF) maturation failure, along with a better understanding of the underlying pathophysiological mechanisms. If our findings confirm this association, routine screening for aPL positivity in patients planned for AVF creation could become a standard practice. This would allow early identification of high-risk individuals, enabling the implementation of preventive measures such as tailored anticoagulation strategies, closer clinical monitoring, or alternative vascular access planning.

This in vitro study has limitations. Cell-cultured conditions differ from physiological conditions, and isolated HMEC-1 cells do not reflect the complex AVF microenvironment composed of different cell types as well as their respective interactions. Cell cultures do not replicate blood flow and shear stress, which are crucial factors in AVF maturation, and cannot reproduce some pathological processes such as stenosis, intimal hyperplasia or thrombosis. Finally, HMEC-1 are microvascular immortalized cells and might limit the interpretation of molecular markers or results observed in an in vitro setting.

## 4. Conclusions

This observational prospective cohort study aims to prospectively examine the association between aPL persistent positivity and native AVF maturation failure in HD patients, potentially identifying antiphospholipid antibody (aPL) persistent positivity as a predictive biomarker for arteriovenous fistula (AVF) maturation failure. Patients’ plasma and serum samples will be collected before AVF creation and before HD initiation in order to further assess the underlying pathophysiological mechanisms. This study was registered on ClinicalTrials.gov (ID number: NCT06112821).

## Figures and Tables

**Table 1:** Baseline characteristics collected at the time of AVF surgical creation.

Parameters	
Demographics	Age
	Gender
	BMI (kg/m^2^)
	Ethnicity
	Smoking status
Treatments	Anti-platelet therapy
	ACE/ARBs
	Statins
	Erythropoietin
	Anticoagulation therapy
	Beta-blockers
Medical history	Etiology of CKD, histologically proven
	Diabetes mellitus
	Hypertension
	Coronary heart disease
	Stroke or TIA
	Ischemic heart disease
	Peripheral vascular disease
	Left ventricular ejection fraction
	Deep vein thrombosis (n, %)
	Other aPL related clinical features
Biological	Hemoglobin level
features	Platelet count, mean platelet volume
	Coagulation tests: activated PTT, fibrinogen, Prothrombine time
	Lipids
	c-reactive protein, ESR
	Nt-proBNP
Vascular access	Need for catheter before AVF use
parameters	AVF creation date, AVF type, surgical procedure information (type of anesthesia, artery and vein diameter, anastomosis diameter)
	Mapping details
	Perioperative complications: bleeding, local hematoma, other complications
	Pre-operative Flow mediated dilation of the brachial artery as a marker of endothelial dysfunction

ACE: angiotensin-converting enzyme inhibitors; ARB: angiotensin II receptor blockers; AVF: arteriovenous fistula; BMI: body mass index; CRP: c-reactive protein; TIA: transient ischemic attack.

**Table 2:** Primary and secondary outcomes.

Primary Outcomes	
AVF maturation	Defined 6 weeks after AVF creation, by US: AVF brachial artery blood flow > 600 mL/min, outflow vein diameter > 6 mm;
	Defined clinically if cannulation is possible with two needles for at least 75% of HD sessions over a continuous 4-week period, including either a mean HD machine blood pump speed superior to 300 mL/min over four consecutive sessions or a measured urea Kt/V > 1.4, or a urea reduction ratio (URR) > 70%
Assisted maturation	Need for any intervention (e.g., ballon angiography, etc.) in order to promote maturation
Time to first AVF puncture	
**Secondary outcomes**	
Primary patency rate	Defined as the time period from AVF creation to the first intervention in order to maintain AVF patency
Functional primary patency rate	Defined as the time period from AVF first cannulation to intervention in order to maintain AVF patency
Thrombosis	Defined as any acute change in AVF physical examination of blood flow leading to a complete inability to perform dialysis.
Stenosis	Defined as chronic change in AVF physical examination of blood flow, associated with a confirmation by using either US or angiography (stenosis of more than 50% in diameter).
Bleeding complications	
HD adequacy	Defined by the Urea Kt/V and Urea Reduction ratio (URR)

## Data Availability

Data are contained within the article.
